# Editorial: Integrating behavioral neuroscience and educational psychology in healthcare training

**DOI:** 10.3389/fmed.2026.1937152

**Published:** 2026-08-13

**Authors:** Sarmishtha Ghosh, Shereen El Tarhouny, Elena Nixon, Tayseer Mansour

**Affiliations:** 1 Adjunct Faculty, Department of Health Professions Education, Parul Institute of Medical Science and Research, Parul University, Vadodara, Gujarat, India; 2 Health Profession Education Center, Ibn Sina National College for Medical Studies, Jeddah, Saudia Arabia; 3 Institute of Mental Health, School of Medicine, University of Nottingham, Nottingham, United Kingdom; 4 Medical Education Department, Faculty of Medicine, Suez Canal University, Ismailia, Egypt; 5 Family and Community Medicine and Medical Education Department, College of Medicine, Taibah University, Madinah, Saudi Arabia

**Keywords:** behavioral, education, healthcare, neuroscience, psychology

Healthcare education is currently navigating an epistemic transformation. For decades, clinical training focused primarily on mastering knowledge and technical skills. Increasingly, educators recognize that this model overlooks how the human brain learns, adapts and sustains performance under the high-pressure demands of contemporary practice. To address this gap, insights from the intersection of behavioral neuroscience and educational psychology are helping to elucidate the cognitive, emotional, and interpersonal substrates of clinical expertise.

This Research Topic, comprising 11 original studies and one conceptual framework, explored how interdisciplinary perspectives can collectively shape the development of well-rounded clinicians. Contributions spanned diverse contexts -including Saudi Arabia, Africa, China, Mexico, Germany, and Egypt- offering a comparative perspective on the evolving landscape of medical education. Three broad themes emerged: the neurocognitive foundations of learning, the development of psychological resilience, and the interpersonal dimensions of professional identity.

## Neurocognitive foundations and learning innovations

A pivotal contribution to this collection was the cognitive ecology of learning. In a study by Fawzi et al., an integrated neurodidactic curriculum was implemented for medical interns in Saudi Arabia. Aligning educational strategies with neuroscientific insights into attention and memory significantly increased cognitive engagement and clinical competency. This work exemplified how multidisciplinary insights can be operationalized within healthcare training to support both learner development and clinical competence.

Extending the exploration of the cognitive ecology of modern learners, Hernández-Chávez et al. showed that excessive screen time, particularly short-form video consumption, correlated negatively with fluid intelligence and academic performance in medical students in Mexico. These findings emphasized that as simulation, e-learning, and digital patient records become the backbone of healthcare training, screen habits should be treated as an element of educational infrastructure element, not a lifestyle issue; hence training environments would benefit from contextualizing and optimizing cognitive ecology rather than fragmenting it.

Pedagogical innovations such as mind mapping and experiential learning also featured prominently in this collection. Cheng et al. and Niu and Ku applied Kolb's experiential learning theory and case-based mind mapping, respectively, to nursing education in China. Both studies demonstrated that structured active learning, aligned with memory consolidation, can enhance theoretical understanding and critical thinking. Both papers provide transferable and theoretically grounded models affirming a fundamental insight from cognitive neuroscience: memory consolidates through active retrieval, emotional engagement, and embodied practice -not passive reception.

## Psychological capital, resilience, and career formation

Another central insight across this collection was that technical skill is inseparable from the learner's psychological resources. Liu et al. demonstrated that psychological capital—i.e., hope, efficacy, resilience, and optimism- mediated the link between work engagement and professional outcomes in nursing interns in China. A key implication of these findings was that the same level of engagement can yield varied developmental outcomes depending on the psychological resources a learner brings to the encounter; hence institutions ignoring their learners' psychological architecture risk optimizing curricular delivery over genuine learning and development.

Teng et al. showed in a group of medical students in Eastern China how early relational environments, influenced by parenting styles, can shape vulnerability to academic burnout, with resilience acting as a protective buffer. The authors pointed out how learners carry attachment histories, dispositional orientations, and family-shaped expectations that influence how they absorb institutional pressure. Treating psychological distress as solely a personal failure while neglecting systemic factors can perpetuate hidden suffering and attrition. Resilience, as this paper affirmed, is a developmental outcome that education can support or undermine.

Zhou et al. developed a Challenge-Resource-Adaptation model to explain how career resilience develops through proactive adaptation and mentor support, utilizing a medical intern group in northwest China. Within this framework, the internship is not merely a period of skill acquisition but a crucible for professional identity that educational systems should treat as a developmental task.

Liang et al. showed that self-efficacy can drive feedback-seeking behavior, mitigating the “transition shock” in Chinese nursing interns. A key implication of this work was that, owing to the brain's plasticity, training conditions can profoundly shape the clinicians who emerge from them.

Collectively, these studies positioned resilience as a trainable competency that can be strengthened through tailored educational scaffolding.

## Interpersonal cognition, values, and the hidden curriculum

This theme addressed how clinicians think and feel both about patients and their professional roles, and the role of the “hidden curriculum” which reflects the implicit values transmitted through clinical culture. Platzer et al. highlighted how personal, social, and contextual factors can shape engagement with sensitive clinical competencies in medical students in Germany. They showed that perceived clinical relevance can drive engagement in sexual-history taking, regardless of biological sex, underscoring the importance of tailoring communication training to diverse motivational profiles.

Moumeni et al. documented a “Rehabilitative Negativity Syndrome” in Central Africa, where medical training amplified therapeutic nihilism toward stroke recovery, highlighting how educational cultures can shape expectations that undermine patient care. Addressing attitudes and cognitive biases within healthcare education was deemed crucial to professional development as the latter involves not only knowledge acquisition but also the formation of beliefs and values that shape clinical practice.

El Tarhouny et al. brought behavioral neuroscience and moral psychology into conversation through their study of emotional intelligence and moral outrage in medical students in Egypt. Emotional regulation mediated by prefrontal-limbic circuitry emerged not as a soft skill supplementary to clinical training but as a structural determinant of ethical behavior under pressure. The authors' assertions indicated how students struggling to regulate moral anger are not morally deficient but neurologically and developmentally under-supported. This paper makes a compelling case for explicit emotional-intelligence training in professional formation.

Finally, El Tarhouny and Farghaly raised the question of what happens to the cognitive architecture of expertise as clinical automation expands. Their conceptual contribution reframes deskilling not merely as a workforce issue but as a neurocognitive one: expertise is encoded through repeated, contextually rich practice; when that practice is offloaded to machines, the neural substrates of competence may atrophy in ways that are invisible until critically needed.

## Concluding remarks

The 12 articles in this Research Topic collectively point toward a fundamental shift in healthcare training- one that places the science of learning at the heart of educational practice. Integrating behavioral neuroscience and educational psychology can enable programs that engage the “whole learner,” encompassing cognitive, emotional, and social development. The call to the global medical community is unequivocal: advancing healthcare requires concurrent attention to brains, minds, and relationships. Sustained, collaborative research will be vital in building on the evidence base for a future workforce defined by intellectual agility, cultural and ethical competence, and transformative capacity within increasingly complex healthcare environments.

